# Information Propagation in Hypergraph-Based Social Networks

**DOI:** 10.3390/e26110957

**Published:** 2024-11-06

**Authors:** Hai-Bing Xiao, Feng Hu, Peng-Yue Li, Yu-Rong Song, Zi-Ke Zhang

**Affiliations:** 1 School of Computer, Qinghai Normal University, Xining 810008, China; xiaohaibing23@gmail.com (H.-B.X.);; 2 The State Key Laboratory of Tibetan Intelligent Information Processing and Application, Xining 810008, China; 3 College of Automation & College of Artificial Intelligence, Nanjing University of Posts and Telecommunications, Nanjing 210023, China; songyr@njupt.edu.cn; 4 Center for Digital Communication Studies, Zhejiang University, Hangzhou 310058, China

**Keywords:** online social networks, hypergraph, information propagation, response process strategies

## Abstract

Social networks, functioning as core platforms for modern information dissemination, manifest distinctive user clustering behaviors and state transition mechanisms, thereby presenting new challenges to traditional information propagation models. Based on hypergraph theory, this paper augments the traditional SEIR model by introducing a novel hypernetwork information dissemination SSEIR model specifically designed for online social networks. This model accurately represents complex, multi-user, high-order interactions. It transforms the traditional single susceptible state (S) into active ($S_{a}$) and inactive ($S_{i}$) states. Additionally, it enhances traditional information dissemination mechanisms through reaction process strategies (RP strategies) and formulates refined differential dynamical equations, effectively simulating the dissemination and diffusion processes in online social networks. Employing mean field theory, this paper conducts a comprehensive theoretical derivation of the dissemination mechanisms within the SSEIR model. The effectiveness of the model in various network structures was verified through simulation experiments, and its practicality was further validated by its application on real network datasets. The results show that the SSEIR model excels in data fitting and illustrating the internal mechanisms of information dissemination within hypernetwork structures, further clarifying the dynamic evolutionary patterns of information dissemination in online social hypernetworks. This study not only enriches the theoretical framework of information dissemination but also provides a scientific theoretical foundation for practical applications such as news dissemination, public opinion management, and rumor monitoring in online social networks.

## 1. Introduction

With the global proliferation and rapid development of the internet, various online social platforms such as QQ, WeChat, Weibo, Twitter, and Facebook have transcended spatial and temporal limitations, becoming essential tools for billions of users worldwide to quickly publish, receive, and share information. These platforms have significantly enhanced the speed and scope of information dissemination, profoundly impacting traditional communication models [1]. Therefore, a systematic study of the mechanisms and patterns of information dissemination within online social networks holds substantial theoretical value and provides crucial guidance for practical applications, such as information monitoring and policy formulation.

Since Goffman and others applied the SIR model to the field of information dissemination in 1964, proposing the use of epidemiological dynamics to study the spread of information among populations [2], a foundation was laid for later models of information dissemination in complex networks. This has had a profound impact, particularly on understanding how information is transmitted through social networks. In 2003, Kleinberg et al. applied disease transmission models to analyze the diffusion of information in social networks and further explored the theoretical mechanisms of information propagation [3], laying the groundwork for studying information dissemination on networks. Subsequently, an increasing number of scholars in the field of information dissemination have introduced epidemiological models such as SIS [4] and SIR [5,6] into social network analysis [7,8], with researchers like Wang proposing a social network information dissemination model based on the SEIR model [9]. As research has deepened, to better reveal the laws of information dissemination on social networks, researchers have proposed models like the H-SEIR model [10], SHIR model [11], and ESIS model [12] to explore the laws of information dissemination. With the enrichment of information dissemination models, a deeper understanding of information dissemination has fostered the analysis of influencing factors, such as memory effects and social reinforcement [13,14,15], node attributes and information value [16,17,18], and node influence [4,19]. In recent years, the scholarly focus has been on complex network models [20,21], deep learning algorithms [22,23], and the analysis and prediction of information dissemination mechanisms [24,25]. In 2017, Li et al. systematically reviewed various information diffusion models proposed over the past decades, discussing their effectiveness in practical applications such as market forecasting and rumor control [26]. In 2022, Guo and others innovated complex network models, proposing the heterogeneous graph attention network [27] using deep attention mechanisms to simulate and predict information paths in heterogeneous networks, demonstrating high predictive accuracy in information propagation. Uthirapathy et al. proposed a method combining fuzzy logic and forest fire algorithms to predict the evolution of user opinions on social networks [28]. In the same year, Iamnitchi et al. conducted an in-depth analysis of manipulative behaviors and the dissemination of false information on social media platforms by proposing a new framework for the analysis of information manipulation, providing better tools for social platforms to monitor and prevent the spread of false information [29]. These models have achieved significant results in describing the basic processes of information dissemination and control.

However, existing research primarily relies on traditional graph structures to construct online social networks [30,31], which are very effective in describing binary relations between single nodes but often fail to capture higher-order interactive dynamics when dealing with complex group characteristics and multiparty information interactions in the network. To overcome these limitations and, more precisely, describe clustering relationships in online social networks, this paper introduces the mathematical tool of hypergraphs. Hypergraphs extend the edges of traditional graphs to connect multiple nodes, offering an effective method to describe multivalent relationships and complex group interactions [32]. The hypergraph model is particularly suited to depicting group interactions and multiparty information exchanges in online social networks, a capability that stems from its structural characteristics, enabling it to naturally map multivalent relationships and high-order interactions [33,34]. Building on the advantages of hypergraphs, this paper further introduces the BA scale-free hypernetwork model, which not only simulates the scale-free characteristics of online social networks [35] but also allows us to explore the clustering behavior of nodes and the heterogeneity of connections through its structure. A prominent feature of scale-free networks is that their degree distribution follows a power law, where a few nodes have many connections while most nodes have few. This structure is widespread in many real-world networks and more accurately reflects the complexity of online social networks; therefore, this paper proposes a novel information dissemination model for online social networks—the SSEIR model. This model expands on the classical SEIR dissemination model by further subdividing the susceptible state (S) into two states: $S_{a}$ (Susceptible Active) and $S_{i}$ (Susceptible Inactive). The $S_{a}$ state represents uninformed but active users, while the $S_{i}$ state represents uninformed and inactive users. This subdivision allows the SSEIR model to more precisely describe the changes in user states within online social networks. Additionally, the SSEIR model, compared to traditional models of information dissemination, includes a direct conversion pathway from the E-state to the R-state and takes into account the conversion of the S-state to different states in the SIR and SEIR models, enriching the rules of state transformation and more accurately reflecting the randomness and complexity of information dissemination in real online social networks. Throughout the theoretical derivation and simulation processes, this paper employs the reaction process (RP) Strategy [4], extensively studying the laws of information propagation in hypernetworks. The research results indicate that the SSEIR model not only excels in fitting actual data but also reveals the intrinsic mechanisms and dynamic evolution of information dissemination.

Section 2 of this paper introduces the concept of hypernetworks, the evolutionary process of the BA scale-free hypernetwork, and the basic principles of traditional information propagation models. Section 3 provides a detailed description of the newly proposed SSEIR model and its theoretical derivation process. Section 4 conducts a simulation analysis of the SSEIR model in the propagation process within social hypernetworks. Section 5 summarizes the main findings of the research and looks forward to future research directions.

## 2. Theoretical Background

### 2.1. Concept of Hypernetworks

Let $V=\left\{v_{1},v_{2},\ldots ,v_{n}\right\}$ and $E=\left\{e_{1},e_{2},\ldots ,e_{m}\right\}$. If $e_{i}\neq \emptyset \left(i=1,2,\cdot \cdot \cdot ,m\right)$ and $\overset{m}{\underset{i=1}{⋃}}e_{i}=V$, then, the binary relation H=(V,E) is called a hypergraph [36]. Here, V is referred to as the set of nodes of the hypergraph and E as the set of edges. An edge $e_{i}=\left\{v_{i1},v_{i2},\cdot \cdot \cdot ,v_{ij}\right\}\;\left(i=1,2,\cdot \cdot \cdot ,m\right)$ is called a hyperedge. If node $v_{i}\;\left(i\leq n\right)$ and $v_{j}\;\left(j\neq i,j\leq n\right)$ belong to the same hyperedge, then nodes $v_{i}$ and $v_{j}$ are said to be adjacent. $\left|e_{i}\right|$ denotes the cardinality of the hyperedge $e_{i}$*,* and if $\left|e_{i}\right|=k\left(i=1,2,\cdot \cdot \cdot ,m\right)$, then H=(V,E) is called a K-uniform hypergraph, also known as a uniform hypergraph. When $\left|e_{i}\right|=2$, the hypergraph H=(V,E) degenerates into an ordinary graph within complex networks. In online social hypernetworks (OSHNs), nodes represent each user, while hyperedges represent the clustering relationships between users, such as friendships, colleague groups, family networks, and community groups.

### 2.2. Construction of OSHN Topological Model

In a hypernetwork, when two or more nodes are included in the same hyperedge, they are considered adjacent. This framework excels at representing the multidimensional relationships among nodes, making it particularly useful for depicting the complex interconnections in online social networks. In this study, hypernetworks are employed to model the clustering relationships among users within these platforms. Drawing on existing research [37,38], this paper proposes a hypernetwork model specifically tailored for simulating online social networks. The construction of this model involves the following steps:

- **Initialization**: Initially, the online social hypernetwork contains $m_{0}$ users, forming a social clustering relationship, where these $m_{0}$ user nodes constitute a single hyperedge;
- **Growth Dynamics**: At each time step t, a new user is added. This user selects $m_{1}$ existing nodes from the hypernetwork to form various new clustering relationships. Consequently, at each time step, m non-repetitive hyperedges are generated in the social hypernetwork;
- **Hyperdegree Preferential Attachment Mechanism**: From the pool of existing nodes in the hypernetwork, $m_{1}$ old nodes are selected to form a new hyperedge with the newly added node based on their hyperdegrees. The probability of selecting a node $v_{i}$ for connection is proportional to the node’s hyperdegree $d_{H}\left(i\right)$, relative to the total hyperdegree $d_{H}\left(j\right)$ of all existing nodes $v_{j}$ in the hypernetwork, expressed as follows:
  (1) $$\prod d_{H}\left(i\right)=\frac{d_{H}\left(i\right)}{\sum_{j}d_{H}\left(j\right)}$$

In the equation, $d_{H}\left(i\right)$ is the hyperdegree of the node $v_{i}$.

The hyperdegree distribution P(k) refers to the proportion of nodes with a hyperdegree of k in the entire network [39]. The hyperdegree distribution of the nodes is independent of time t. After a period of evolution, P(k) is as follows:

(2) $$P\left(k\right)=\frac{1}{m}\cdot \left(\frac{1}{m_{1}}+1\right)\cdot {\left(\frac{m}{k}\right)}^{\frac{1}{m_{1}}+2}$$

**Figure 1:**
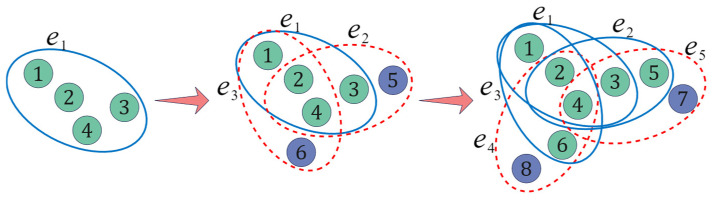
Evolutionary schematic of the hypernetwork model (m=2; $m_{1}=3$). Blue solid lines indicate existing hyperedges, green nodes denote existing nodes, red dashed lines depict new hyperedges added in the current time step, and blue nodes signify new nodes added during the current time step.

In real social networks, with the increase in time steps, a new user who joins may opt to establish m groups, each comprising $m_{1}$ existing nodes, thus forming new clustering relationships. This evolutionary process is depicted in Figure 1.

### 2.3. Traditional Propagation Model: SEIR

The Susceptible-Exposed-Infected-Recovered (SEIR) model is an advanced extension of the SIR model, incorporating an E-state to account for an incubation period [9]. In this model, individuals pass through four stages: susceptible (S), exposed (E), infected (I), and recovered (R). Susceptible individuals, upon contact with infected ones, may enter the exposed state (E), which represents an incubation period, but not all contacts result in transmission. This transition depends on specific conditions of transmissibility and susceptibility, reflecting the probabilistic nature of infection. After this period, they progress to the infected state, where they actively spread the infection and ultimately recover, acquiring permanent immunity in the R-state.

**Figure 2:**
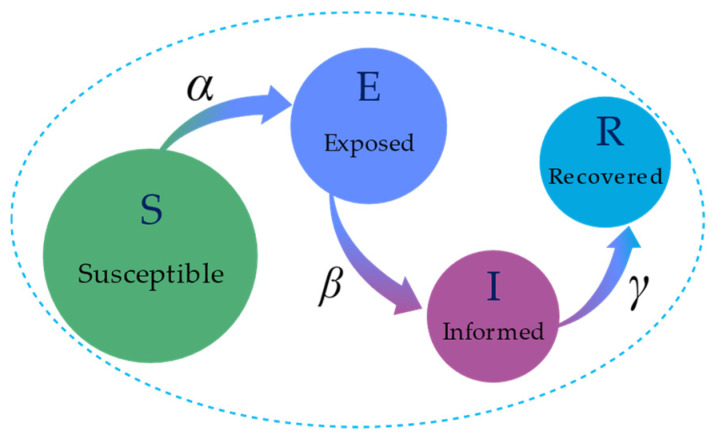
SEIR model state transition diagram. In the context of information dissemination, the green section represents the S-state, indicating unawareness of the information. The dark blue section is the E-state, where individuals are aware of but not spreading the information. The purple section denotes the I-state, where individuals actively spread the information. The light blue section represents the R-state, indicating immunity to the information.

In the context of information propagation, the SEIR model is particularly effective in describing scenarios where there is a discernible lag between receiving and disseminating information. Specifically, individuals receive information and transition from the S-state to the E-state, during which they do not spread the information. Following the exposed period, they begin to actively disseminate the information as they move from the E-state to the I-state. Eventually, they lose interest or relevance in the information, ceasing its spread and transitioning to the R-state. This model is apt for scenarios involving scientific research findings, technological innovations, and complex social issues, where the dissemination process includes significant time lags. The state transition dynamics of the SEIR model is illustrated in Figure 2.

Here, α represents the probability that an S-state user will convert to an E-state user after contact with an I-state user, β represents the probability that an E-state user will convert to an I-state user at each time step, and γ represents the probability that an I-state user will convert to an R-state user at each time step. The differential dynamic equations are as follows:

(3) $$\left\{\begin{array}{c} \frac{dS\left(t\right)}{dt}=-\alpha I\left(t\right)S\left(t\right)\\ \frac{dE\left(t\right)}{dt}=\alpha I\left(t\right)S\left(t\right)-\beta E(t)\\ \frac{dI\left(t\right)}{dt}=\beta E\left(t\right)-\gamma I(t)\\ \frac{dR\left(t\right)}{dt}=\gamma I\left(t\right) \end{array}\right.$$

## 3. Proposal of the New Model

### 3.1. Shortcomings of Traditional Models

Although the SIS, SIR, and SEIR models have achieved some success in the study of information propagation, there are still significant shortcomings when applied to information dissemination in online social networks.

Firstly, the diversity of user behavior is overlooked. Traditional models typically simplify individual states into basic types such as susceptible, informed, and recovered. However, in online social networks, user behavior is highly diverse and complex. Users differ in their levels of activity and engagement when receiving and spreading information, and traditional models struggle to capture these differences.

Secondly, there is a failure to refine the S-state. In traditional models, the S-state is often treated as a single state. However, in online social networks, the S-state of users can be further subdivided. For example, some users may be unaware of certain information but are highly active online and likely to receive new information, whereas others are less active and less likely to receive new information. Traditional models have difficulty distinguishing between these two types of users, leading to a lack of precision in the model.

Lastly, traditional models ignore the dynamic changes of the exposed period. Although the SEIR model introduces an E-state, in practice, the duration and characteristics of the exposed period can vary depending on the type of information and user behavior. Models typically assume a fixed exposed period, presuming that users will definitely enter this phase. However, this is not always applicable in the complex environment of online social networks, limiting the flexibility and accuracy of the model.

### 3.2. Proposal of the SSEIR Model

This model classifies susceptible users based on their activity levels in online social networks into two categories: active ($S_{a}$) and inactive ($S_{i}$), alongside the traditional states: exposed (E), informed (I), and recovered (R). The $S_{a}$-state includes active users who are yet to be aware of the information, while the $S_{i}$-state includes inactive users also unaware of the information. In the E-state, users know the information but have not started spreading it. They might move to the I-state to start dissemination or directly to the R-state to cease spreading. The I-state applies to users who are aware of the information and are actively spreading it on online social networks. The R-state refers to users who are aware of the information and no longer spread it.

The SSEIR model’s state transitions are defined by specific probabilities that mirror the dynamics of how information spreads in online social networks:

- From $S_{i}$ to E: A node adjacent to an I-state node moves to E-state with a probability of α;
- From $S_{a}$ to I: A node adjacent to an I-state node shifts to I-state with a probability of β, starting to disseminate information;
- From E to I or R: Nodes in that E-state have a θ probability of moving to I-state to spread information or a γ probability of going directly to R-state to stop spreading;
- From I to R: Nodes in the I-state move to the R-state with a probability of ε, ending their role in information dissemination. Additionally, the probability v accounts for forgetting information, leading to R-state.

**Figure 3:**
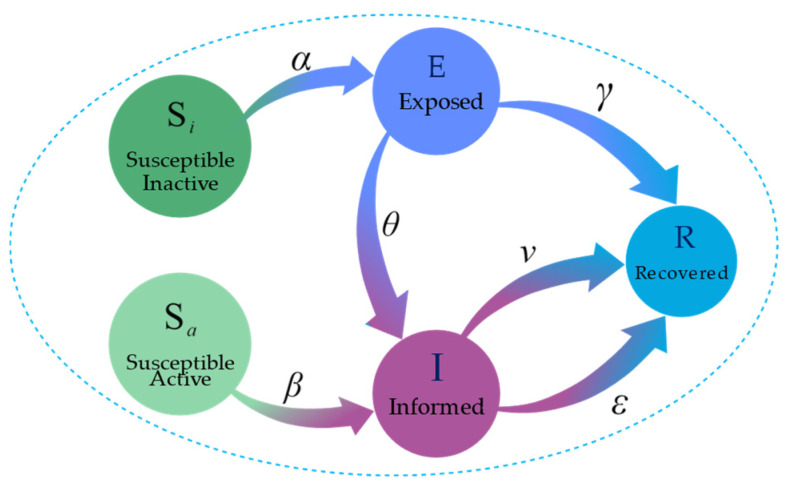
SSEIR model state transition diagram. Dark green denotes the $S_{i}$-state, light green denotes the $S_{a}$-state, dark blue denotes the E-state, purple denotes the I-state, and light blue denotes the R-state.

Our proposed model comprehensively considers the simplified scenario in the traditional SIR model where individuals transition directly from the S-state to I-state, as well as the process in the SEIR model where individuals transition from the S-state to E-state, then to the I-state. For highly active susceptible individuals in social networks, namely those in the $S_{a}$-state, we observe that they can almost immediately transition to I-state upon encountering information. Therefore, for simplicity of the model, we assume that these $S_{a}$-state individuals transition directly to the I-state without passing through the E-state upon receiving information. This assumption simplifies the complexity of the model while effectively capturing the rapid dissemination of information among highly active individuals. All these dynamic state transitions are detailed in the state transition diagram of our SSEIR model (Figure 3).

When information is propagated through the network, for a user node i in the social network, its state may transition between $S_{i}$, $S_{a}$, E, I, and R. During the time interval $\left[t,t+∆t\right]$, the probability of node i undergoing a state transition is as shown in Table 1.

To explore the differential dynamic equations of the SSEIR model in social hypernetworks, the following derivation is conducted.

Assuming that at the time t, the total number of nodes with K adjacent nodes in the network are $N\left(K,t\right)$, then the following occurs:

(4) $$S_{i}\left(K,t\right)+S_{a}\left(K,t\right)+E\left(K,t\right)+I\left(K,t\right)+R\left(K,t\right)=N\left(K,t\right)$$

During the time interval $\left[t,t+∆t\right]$, the changes in the number of nodes of each state are as follows:

(5) $$S_{i}\to S_{i}\left(K,t+∆t\right)=S_{i}\left(K,t\right)\cdot \bar{P_{S_{i}S_{i}}\left(K,t\right)}$$

(6) $$S_{a}\to S_{a}\left(K,t+∆t\right)=S_{a}\left(K,t\right)\cdot \bar{P_{S_{a}S_{a}}\left(K,t\right)}$$

(7) $$E\to E\left(K,t+∆t\right)=E\left(K,t\right)+S_{i}\left(K,t\right)\cdot \left[1-\bar{P_{S_{i}S_{i}}\left(K,t\right)}\right]-E\left(K,t\right)\cdot \left(P_{EI}+P_{ER}\right)$$

(8) $$\begin{array}{ll} I\to I\left(K,t+∆t\right) & =I\left(K,t\right)+S_{a}\left(K,t\right)\cdot \left[1-\bar{P_{S_{a}S_{a}}\left(K,t\right)}\right]+E\left(K,t\right)\cdot P_{EI}-I\left(K,t\right)\\ & \cdot \bar{P_{IR}\left(K,t\right)} \end{array}$$

(9) $$R\to R\left(K,t+∆t\right)=R\left(K,t\right)+E\left(K,t\right)\cdot P_{ER}+I\left(K,t\right)\cdot \bar{P_{IR}\left(K,t\right)}$$

To derive the average rates of change for each state, we perform the following calculations, starting with the derivation of the average probability that nodes in $S_{i}$-state with K adjacent nodes at the time t remain in the same state, denoted as $\bar{P_{S_{i}S_{i}}\left(K,t\right)}$.

Assuming node i is in $S_{i}$-state at time t, then the following occurs:

(10) $$P_{S_{i}S_{i}}^{i}+P_{S_{i}E}^{i}=1$$

At time t, the number of neighboring nodes of the node i that are in I-state is $X_{I}=X_{I}\left(t\right)$; thus, the following occurs:

(11) $$P_{S_{i}S_{i}}^{i}={\left(1-∆t\cdot \alpha \right)}^{X_{I}}$$

Assuming the hyperdegree of node i is k, the number of neighboring nodes for this node is K:

(12) $$K=k\cdot m_{1}$$

According to mean field theory, combined with Equation (2), the average hyperdegree 〈k〉 is calculated:

(13) $$\langle k\rangle =\int_{m}^{\infty }k\cdot P\left(k\right)dk=m\cdot m_{1}$$

$X_{I}$ is a random variable that follows a binomial distribution:

∏XI,t=K XIαK,tXI·1−αK,tK−XI

In the above equation, $\alpha \left(K,t\right)$ represents the probability at a time t that an active state node with K neighboring nodes is adjacent to an exposed node in the I-state. $p\left(K_{1}|K\right)$ is defined as the neighbor relation function, which represents the probability that a node with K neighboring nodes is adjacent to a node with $K_{1}$ neighboring nodes. $p\left(I_{K_{1}}|S_{K}\right)$ is defined as the probability that a node with $K_{1}$ neighboring nodes are in the I-state, given it is connected to a node with K neighboring nodes in an inactive state:

$$\alpha \left(K,t\right)=\sum_{K_{1}}p\left(K_{1}|K\right)\cdot p\left(I_{K_{1}}|S_{K}\right)$$

$p^{I}\left(K_{1},t\right)$ is used to represent the density of informed nodes with $K_{1}$ neighboring nodes at a time t:

(14) $$\alpha \left(K,t\right)=\sum_{K_{1}}p\left(k_{1}|k\right)\cdot p^{I}\left(K_{1},t\right)$$

The average probability that a node with K neighboring nodes remain in the $S_{i}$-state during the time interval $\left[t,t+∆t\right]$, is denoted as $\bar{P_{S_{i}S_{i}}\left(K,t\right)}$:

(15) PSiSiK,t¯=∑XI=0KKXI1−α·∆tXI·αK,tXI·1−αK,tK−XI=1−α·∆t·αK,tK

By substituting Equation (16) into Equation (17), we obtain the following:

(16) $$\bar{P_{S_{i}S_{i}}\left(K,t\right)}={\left[1-\alpha \cdot ∆t\cdot \sum_{K_{1}}p\left(K_{1}|K\right)\cdot p^{I}\left(K,t\right)\right]}^{K}$$

By substituting Equation (18) into Equation (12), we obtain the following:

(17) $$\bar{P_{S_{i}E}\left(K,t\right)}=1-\bar{P_{S_{i}S_{i}}\left(K,t\right)}=1-{\left[1-\alpha \cdot ∆t\cdot \sum_{K_{1}}p\left(K_{1}|K\right)\cdot p^{I}\left(K,t\right)\right]}^{K}$$

When node i is in the $S_{a}$-state at time t, derive the average probability that nodes in $S_{a}$-state with K adjacent nodes at a time t remain in the same state, denoted as $\bar{P_{S_{a}S_{a}}\left(K,t\right)}$. Similarly, we can obtain the following:

(18) $$\bar{P_{S_{a}S_{a}}\left(K,t\right)}={\left[1-\beta \cdot ∆t\cdot \sum_{K_{1}}p\left(K_{1}|K\right)\cdot p^{I}\left(K,t\right)\right]}^{K}$$

At time t, when node i is in the E-state, the analysis focuses on the probability of state transition for nodes in the E-state over the time interval ∆t:

(19) $$P_{EE}^{i}+P_{ER}^{i}+P_{EI}^{i}=1$$

(20) $$\left\{\begin{array}{c} P_{EI}^{i}=∆t\cdot \theta \\ P_{ER}^{i}=∆t\cdot \gamma \\ P_{EE}^{i}=1-∆t\cdot \theta -∆t\cdot \gamma \end{array}\right.$$

If node i is in the I-state at a time t, the rate of change for nodes in the I-state over the time interval ∆t is analyzed:

(21) $$P_{II}^{i}+P_{IR}^{i}=1$$

(22) $$\left\{\begin{array}{c} P_{IR}^{i}=∆t\cdot \left(v+\varepsilon \right)\\ P_{II}^{i}=1-∆t\cdot \left(v+\varepsilon \right) \end{array}\right.$$

For nodes with K adjacent nodes in the $S_{i}$-state, the average state retention probability derived from Equation (16) into Equation (5) is inserted to illustrate their numerical changes over the interval ∆t:

(23) $$\begin{array}{ll} S_{i}\left(K,t+∆t\right) & =S_{i}\left(K,t\right)\cdot \bar{P_{S_{i}S_{i}}\left(K,t\right)}\\ & =S_{i}\left(K,t\right)\cdot {\left[1-\alpha \cdot ∆t\cdot \sum_{K_{1}}p\left(K_{1}|K\right)\cdot p^{I}\left(K,t\right)\right]}^{K} \end{array}$$

For nodes with K adjacent nodes in the $S_{a}$-state, incorporating Equation (18) into Equation (6) provides their numerical changes over the interval ∆t:

(24) $$\begin{array}{ll} S_{a}\left(K,t+∆t\right) & =S_{a}\left(K,t\right)\cdot \bar{P_{S_{a}S_{a}}\left(K,t\right)}\\ & =S_{a}\left(K,t\right)\cdot {\left[1-\beta \cdot ∆t\cdot \sum_{K_{1}}p\left(K_{1}|K\right)\cdot p^{I}\left(K,t\right)\right]}^{K} \end{array}$$

For nodes with K adjacent nodes in the E-state, incorporating Equations (16) and (20) into Equation (7) provides the numerical changes after the time interval ∆t:

(25) $$\begin{array}{ll} E\left(K,t+∆t\right) & =E\left(K,t\right)+S_{i}\left(K,t\right)\cdot \left[1-\bar{P_{S_{i}S_{i}}\left(K,t\right)}\right]-E\left(K,t\right)\cdot \left(P_{EI}+P_{ER}\right)\\ & =E\left(K,t\right)+S_{i}\left(K,t\right)\cdot \left\{1-{\left[1-\alpha \cdot ∆t\cdot \sum_{K_{1}}p\left(K_{1}|K\right)\cdot p^{I}\left(K,t\right)\right]}^{K}\right\}-E\left(K,t\right)\\ & \cdot \left(\theta +\gamma \right)\cdot ∆t \end{array}$$

For nodes with K adjacent nodes in the I-state, the inclusion of Equations (18), (20), and (22) into Equation (8) provides their numerical changes after the time interval ∆t:

(26) $$\begin{array}{ll} I\left(K,t+∆t\right) & =I\left(K,t\right)+S_{a}\left(K,t\right)\cdot \left[1-\bar{P_{S_{a}S_{a}}\left(K,t\right)}\right]+E\left(K,t\right)\cdot P_{EI}-I\left(K,t\right)\cdot P_{IR}\\ & =I\left(K,t\right)+S_{a}\left(K,t\right)\cdot \left\{1-{\left[1-\beta \cdot ∆t\cdot \sum_{K_{1}}p\left(K_{1}|K\right)\cdot p^{I}\left(K,t\right)\right]}^{K}\right\}+E\left(K,t\right)\\ & \cdot \theta \cdot ∆t-I\left(K,t\right)\cdot \left(v+\varepsilon \right)\cdot ∆t \end{array}$$

For nodes with K adjacent nodes in R-state, Equations (20) and (22) are substituted into Equation (9) to determine the change in their number after a time interval ∆t:

(27) $$\begin{array}{ll} R\left(K,t+∆t\right) & =R\left(K,t\right)+E\left(K,t\right)\cdot P_{ER}+I\left(K,t\right)\cdot P_{IR}\\ & =R\left(K,t\right)+E\left(K,t\right)\cdot ∆t\cdot \gamma +I\left(K,t\right)\cdot \left(v+\varepsilon \right)\cdot ∆t \end{array}$$

For nodes in $S_{i}$-state, the rate of change in their quantity over the time interval ∆t is as follows:

(28) $$\begin{array}{ll} \frac{S_{i}\left(K,t+∆t\right)-S_{i}\left(K,t\right)}{N\left(K,t\right)\cdot ∆t} & =\frac{\partial p^{S_{i}}\left(K,t\right)}{\partial t}\\ & =-p^{S_{i}}\left(K,t\right)\cdot K\cdot \alpha \cdot \sum_{K_{1}}p\left(K_{1}|K\right)\cdot p^{I}\left(K,t\right) \end{array}$$

For nodes in $S_{a}$-state, the rate of change in their quantity over the time interval ∆t is as follows:

(29) $$\begin{array}{ll} \frac{S_{a}\left(K,t+∆t\right)-S_{a}\left(K,t\right)}{N\left(K,t\right)\cdot ∆t} & =\frac{\partial p^{S_{a}}\left(K,t\right)}{\partial t}\\ & =-p^{S_{a}}\left(K,t\right)\cdot K\cdot \beta \cdot \sum_{K_{1}}p\left(K_{1}|K\right)\cdot p^{I}\left(K,t\right) \end{array}$$

For nodes in E-state, the rate of change in their quantity over the time interval ∆t is as follows:

(30) $$\begin{array}{ll} \frac{E\left(K,t+∆t\right)-E\left(K,t\right)}{N\left(K,t\right)\cdot ∆t} & =\frac{\partial p^{E}\left(K,t\right)}{\partial t}\\ & =-p^{E}\left(K,t\right)\cdot \left(\theta +\gamma \right)+p^{S_{i}}\left(K,t\right)\cdot K\cdot \alpha \cdot \sum_{K_{1}}p\left(K_{1}|K\right)\cdot p^{I}\left(K,t\right) \end{array}$$

For nodes in I-state, the rate of change in their quantity over the time interval ∆t is as follows:

(31) $$\begin{array}{ll} \frac{I\left(K,t+∆t\right)-I\left(K,t\right)}{N\left(K,t\right)\cdot ∆t} & =\frac{\partial p^{I}\left(K,t\right)}{\partial t}\\ & =-p^{I}\left(K,t\right)\cdot \left(\varepsilon +v\right)+p^{E}\left(K,t\right)\cdot \theta +p^{S_{a}}\left(K,t\right)\cdot K\cdot \beta \cdot \sum_{K_{1}}p\left(K_{1}|K\right)\\ & \cdot p^{I}\left(K,t\right) \end{array}$$

For nodes in R-state, the rate of change in their quantity over the time interval ∆t is as follows:

(32) $$\frac{R\left(K,t+∆t\right)-R\left(K,t\right)}{N\left(K,t\right)\cdot ∆t}=\frac{\partial p^{R}\left(K,t\right)}{\partial t}=p^{I}\left(K,t\right)\cdot \left(\varepsilon +v\right)+p^{E}\left(K,t\right)\cdot \gamma$$

$S_{i}$, $S_{a}$, E, I, and R are used to respectively represent $p^{S_{i}}\left(K,t\right)$, $p^{S_{a}}\left(K,t\right)$, $p^{I}\left(K,t\right)$, $p^{I}\left(K,t\right)$, and $p^{R}\left(K,t\right)$, and denote the neighbor relation function as $\sum_{K_{1}}p\left(K_{1}|K\right)$ by de. Therefore, Equations (28)–(32) are simplified and substituted into Equations (12) and (13) to derive the following differential dynamic equations for each state:

(33) $$\left\{\begin{array}{c} \frac{dS_{i}}{dt}=-S_{i}\cdot m\cdot {m_{1}}^{2}\cdot \alpha \cdot de\cdot I\\ \frac{dS_{a}}{dt}=-S_{a}\cdot m\cdot {m_{1}}^{2}\cdot \beta \cdot de\cdot I\\ \frac{dE}{dt}=-\left(\theta +\gamma \right)\cdot E+S_{i}\cdot m\cdot {m_{1}}^{2}\cdot \alpha \cdot de\cdot I\\ \frac{dI}{dt}=-\left(\varepsilon +v\right)\cdot I+E\cdot \theta +S_{a}\cdot m\cdot {m_{1}}^{2}\cdot \beta \cdot de\cdot I\\ \frac{dR}{dt}=\left(\varepsilon +v\right)\cdot I+\gamma \cdot E \end{array}\right.$$

## 4. Simulation Results and Analysis

This section compares theoretical and simulation results, aiming to validate that the newly proposed SSEIR model, when combined with hypernetworks, fits better than combinations with ordinary networks and other traditional models. Based on the construction algorithm described in Section 2.2, an online social hypernetwork model was constructed. The new information propagation model was applied to this newly built hypernetwork to explore its superiority over complex networks and to analyze how network topology and state transition rates affect information propagation. To eliminate the influence of randomness, each set of simulation experiments was conducted under the same initial conditions and was independently repeated 50 times to obtain average results.

### 4.1. Theoretical and Simulation Results Comparison

**Figure 4:**
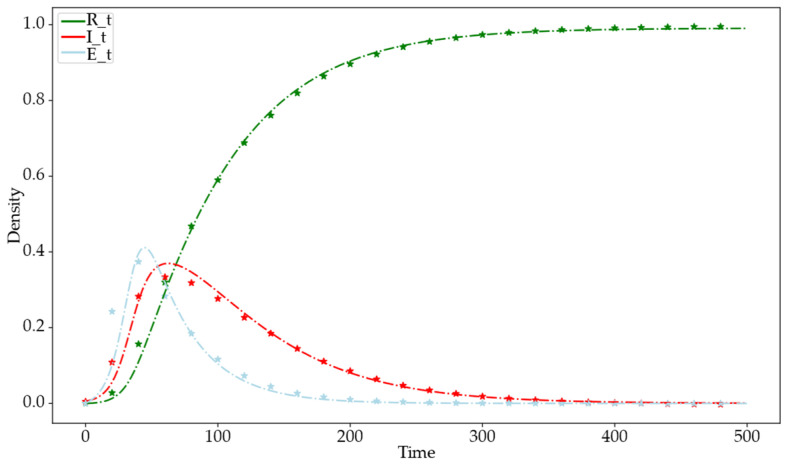
Comparison chart of theoretical and simulation trends in information dissemination. The green dashed line represents theoretical values for the R-state, the red dashed line for the I-state, and the light blue dashed line for the E-state. Green star-shaped markers denote simulation results for the R-state, red stars for the I-state, and light blue stars for the E-state.

To verify the accuracy of the differential dynamic equations derived in the theoretical section, the parameters are set as follows: N=1000, $S_{i}:S_{a}=7:3$, m=2, $m_{1}=5$, de=0.05, α=0.9, β=0.8, θ=0.018, γ=0.01, ε=0.008, and v=0.008. Figure 4 shows a comparison of the information propagation trends between theoretical and simulation experiments. The x-axis represents time steps, and the y-axis represents the density of nodes in each state at each time step. In the figure, continuous lines represent theoretical values, and symbols mark the average state densities at each time step across 50 simulation experiments. The comparison between the theoretical curves and the simulation experiments shows that the theoretical results align with the simulation results, confirming the accuracy of the information propagation differential dynamic equations.

### 4.2. Dynamics Simulation Analysis of Different Network Structures

To verify that hypergraphs more effectively reflect the clustering relationships among users in online social networks compared to ordinary complex networks, information propagation was analyzed using hypergraphs, BA scale-free networks, and NW small-world networks as the underlying networks. This study examined the trends in the density of nodes in different states over time. The theoretical experimental parameters set using mean field theory are as follows: N=1000, $S_{i}:S_{a}=7:3$, de=0.05, α=0.9, β=0.8, θ=0.018, γ=0.01, ε=0.008, v=0.008, m=2, and $m_{2}=5$. The simulation experimental parameters are set as follows: N=1000, $S_{i}:S_{a}=7:3$, α=0.0125, β=0.008, θ=0.03, γ=0.02, ε=0.02, and v=0.01.

**Figure 5:**
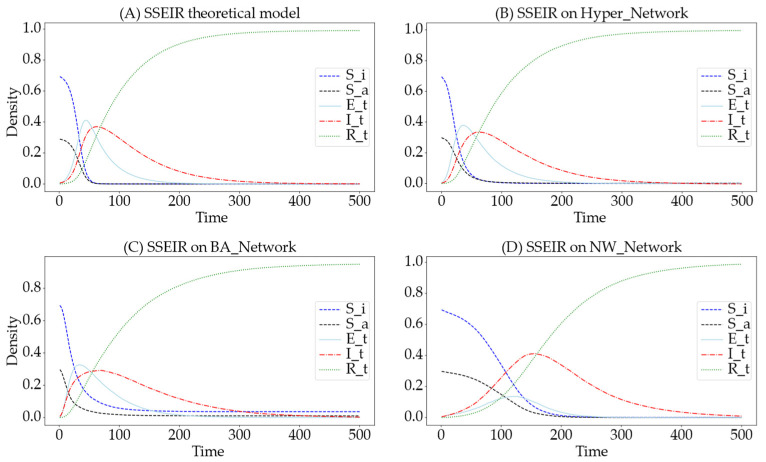
Trends of information dissemination across different network models. Deep blue denotes the $S_{i}$-state, black denotes the $S_{a}$-state, light blue denotes the E-state, red denotes the I-state, and green denotes the R-state. (**A**) displays the theoretical curves of the model, (**B**) applies the model to a hypernetwork, (**C**) to a BA scale-free network, and (**D**) to an NW small-world network.

From the analysis of Figure 5 comparing the information propagation trends under theoretical propagation models, NW, BA, and hypernetwork structures, the conclusions are as follows.

The trend of I(t): The change in the number of informed in the NW small-world network structure shows a trend similar to that of a normal distribution. In the BA scale-free network and hypergraph-based hypernetwork structures, the change in the number of informed exhibits a trend of rapid increase followed by a slow decrease, which is roughly consistent with the theoretical values of the informed. The trend in the NW small-world network differs significantly from the theoretical values because the node degrees in the small-world network do not exhibit a power-law distribution.

The peak of R(t): In simulation scenarios, we observed that for the NW small-world and hypernetwork structures, the proportion of recovered individuals approaches approximately 0.99 after reaching equilibrium, while in the BA scale-free network, this proportion approaches approximately 0.95. These results suggest potential differences in how these network structures might support information dissemination, possibly due to the BA scale-free network’s less effective representation of clustering, a key feature of many real-world social networks. It should be noted, however, that these values are derived from model simulations and may reflect idealized scenarios. Actual observations in real-world settings might encounter challenges in measuring such precise differences due to practical limitations in data collection and variability in network behavior.

Time to reach the peak: In the NW small-world network, the number of informed reaches its peak at time step 150. In contrast, in the BA scale-free network, it peaks earlier at step 65, and in the hypernetwork, even sooner at time step 55. Initially, the curve representing the number of informed in both the hypernetwork and the BA scale-free network exhibits a rapid increase. However, the curve in the BA scale-free network does not accurately capture the initial fermentation process of information typical in social networks. On the other hand, the informed curve in the hypernetwork depicts a more realistic pattern with a slow rise followed by a rapid outbreak, which aligns more closely with the curve of theoretical values.

In conclusion, the underlying hypernetwork model based on hypergraphs more accurately reflects the clustering relationships among users in online social networks and demonstrates clear advantages over ordinary complex networks. Based on these experimental findings, subsequent simulation experiments will be conducted on the hypergraph-based models to further explore their robustness and efficacy in mimicking real-world social dynamics. This continued research will aim to refine our understanding of how information propagates through complex network structures and to develop more effective strategies for information management and dissemination in digital environments.

### 4.3. The Impact of Spreading Rate θ on Information Propagation

In the SSEIR model, inactive users who receive information become exposed (E) users. When users are in the E-state, there is a certain probability that they will transition to either the I-state or the R-state. In social networks, the status transitions of users in this E-state often determines the direction of public opinion trends. Therefore, exploring the impact of the θ on information propagation is crucial. In this experiment, the simulation parameters are set as follows: N=1000, $S_{i}:S_{a}=7:3$, α=0.0125, β=0.008, γ=0.02, ε=0.02, v=0.01, with θ values set at 0.01, 0.03, and 0.05, respectively.

**Figure 6:**
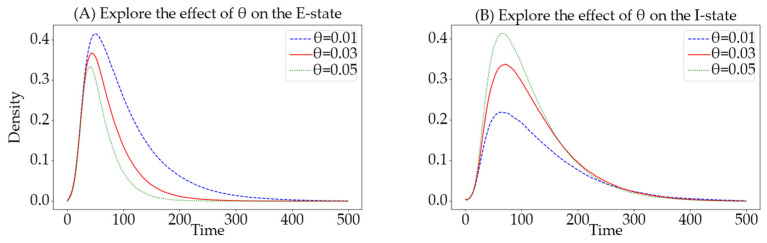
Impact of different θ on the quantities of I-state and E-state. The (**A**) displays the effect on the I-state, while the (**B**) shows the effect on the E-state. The green curve corresponds to a spreading rate of 0.005, the red to 0.03, and the blue to 0.05.

By comparing the different θ, the curves representing the number of informed and the number of exposed individuals are plotted, as shown in Figure 6. The green curve represents θ=0.05, the red curve represents θ=0.03, and the blue curve represents θ=0.01. Observations from the graph indicate that as the θ increases, not only is the peak of the number of informed higher, but the number of exposed users at each time step before reaching a steady state also decreases, while the time to reach this peak remains constant.

In summary, the analysis of the curves for the number of informed and exposed individuals suggests that a higher θ leads to more individuals participating in the spread of information in social networks and fewer individuals remaining in the undecided E-state. This demonstrates the impact of the spreading rate on the dynamics of information spread within social networks.

### 4.4. The Impact of Recovering Rate ε on Information Propagation

In the SSEIR model, users in the I-state recover to the R-state with a probability ε and also transition to the R-state at a rate v due to forgetting information. In social networks, users forgetting information is only influenced by time. Therefore, to control the duration that users remain in the I-state, it is necessary to analyze and explore the ε. In this experiment, the simulation parameters are set as follows: N=1000, $S_{i}:S_{a}=7:3$, α=0.0125, β=0.008, γ=0.02, θ=0.03, v=0.01, with ε values set at 0.01, 0.02, and 0.04, respectively.

**Figure 7:**
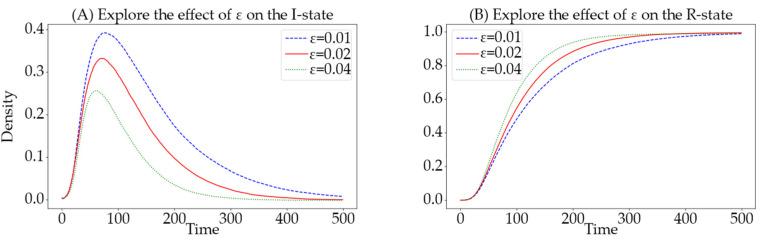
Effects of different ε on the quantities of I-state and R-state. The (**A**) shows the effect of recovering rate on the I-state, while the (**B**) details the effect on the R-state. The green curve indicates a ε of 0.04, the red a rate of 0.02, and the blue a rate of 0.01.

By analyzing the different ε, we plotted the curves for the number of informed and the number of recovered individuals, as shown in Figure 7. The green curve represents ε=0.04, the red curve represents ε=0.02, and the blue curve represents ε=0.01. Observations from Figure 7 indicate that as the ε increases, the peak number of informed decreases, and the time required for the number of informed to reduce to zero also diminishes with each subsequent time step. Furthermore, an increase in the ε leads to a quicker convergence of individuals reaching the R-state to 1. In summary, the analysis of the curves for the number of informed and recovered individuals suggests that a higher ε results in fewer informed within the social network and accelerates the stabilization of the network as a whole.

### 4.5. The Impact of the Number of Adjacent Nodes K on Information Propagation

In the process of information propagation, we simulate an online social network using a hypergraph-based hypernetwork, where nodes represent users in the social network. The more adjacent nodes a node has, the more directly connected individuals a user has in the social network. The greater the average number of adjacent nodes across the network, the closer the relationships among users in the network, which may facilitate information propagation. Therefore, to explore the impact of the number of adjacent nodes on information propagation, we change the parameters m and $m_{1}$ used during network model construction. According to mean field theory, the average number of adjacent nodes in the network is $k=m\cdot m_{1}$. In this experimental process, the simulation parameters are set as follows: N=1000, $S_{i}:S_{a}=7:3$, α=0.0125, β=0.008, γ=0.02, θ=0.03, ε=0.02, and v=0.01.

**Figure 8:**
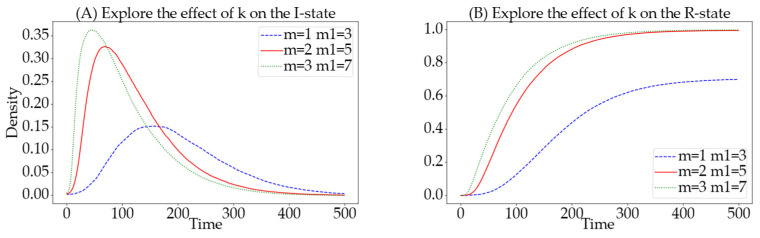
Impact of varying average numbers of adjacent nodes on the quantities of I-state and R-state. The (**A**) details the effects on the I-state, while the (**B**) details the effects on the R-state. The green curve denotes m=3, $m_{1}=7$; the red curve denotes m=2, $m_{1}=5$; the blue curve denotes m=1, $m_{1}=3$.

We conducted comparative experiments with configurations of $m=1,m_{1}=3$, $m=2,m_{1}=5$, and $m=3,m_{1}=7$ creating different network structures with varying average numbers of adjacent nodes. In the resulting plots, the green curve represents the average number of adjacent nodes k=21, the red curve represents k=10, and the blue curve represents k=3. Observations from Figure 8 indicate that networks with a higher average number of adjacent nodes involve more individuals in information propagation, facilitating faster and more widespread dissemination of information, as evidenced by quicker peaks in the number of informed. Furthermore, as the average number of adjacent nodes increases, there is a corresponding increase in the number of individuals reaching the R-state at the same time step, indicating that more people in the network receive information when the average number of adjacent nodes is higher. In conclusion, through comparative experiments on different average numbers of adjacent nodes, it is evident that a higher average number of adjacent nodes in the network enhances the effectiveness of information propagation.

### 4.6. Impact of the Ratio of Active to Inactive Users on Information Propagation

In the newly proposed SSEIR model of this paper, susceptible users are divided into active and inactive users, which effectively simulate the activity habits of users in online social networks. In a network, the ratio of these two types of susceptible users affects the trend of information propagation, and in real social networks, platforms with a higher proportion of active users are preferred for information dissemination. Therefore, exploring the ratio of active to inactive users has significant research value for controlling information propagation. In this experimental process, the simulation parameters are set as follows: N=1000, α=0.0125, β=0.008, γ=0.02, θ=0.03, ε=0.02, and v=0.01.

**Figure 9:**
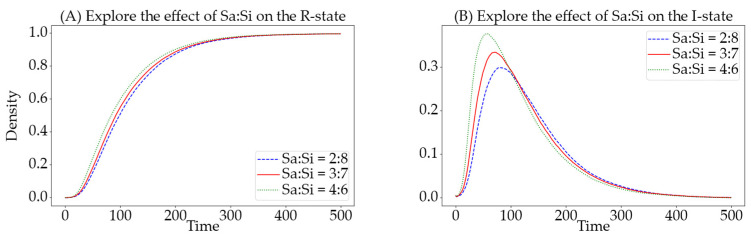
Impact of different ratios of active ($S_{a}$) to inactive ($S_{i}$) nodes on the quantities of I-state and R-state. The (**A**) details the effect on the R-state, while the (**B**) details the effect on the I-state. The green curve indicates a ratio of $S_{a}:S_{i}=4:6$, the red a ratio of $S_{a}:S_{i}=3:7$, and the blue a ratio of $S_{a}:S_{i}=2:8$.

We depicted the ratios $S_{a}:S_{i}=2:8$ with a blue curve, $S_{a}:S_{i}=3:7$ with a red curve, and $S_{a}:S_{i}=4:6$ with a green curve. Observations from Figure 9 reveal that a higher proportion of active users ($S_{a}$) leads to a larger peak in the number of informed and a quicker time to reach this peak. Furthermore, as the proportion of active users increases, the time required for the number of users in the R-state to reach its peak decreases, leading to a quicker stabilization of the system. In summary, a higher proportion of active users in the population significantly enhances the efficiency of information dissemination.

### 4.7. Comparison of SIR, SEIR, and SSEIR Propagation Models

In online social networks, the characteristics of information dissemination typically involve rapid outbreaks followed by swift declines. Although traditional models of information dissemination, such as SIR and SEIR, are derived from viral transmission models and are applicable to information spread, they often overlook the specific rapid burst and fade dynamics characteristic of online social networks. In online social networks, users in the active E-state and I-state significantly impacts the spread of information. Therefore, accurately simulating the trajectory of active users is crucial in assessing model performance. This experiment compares the accuracy of the SIR, SEIR, and newly proposed SSEIR models in simulating changes in active users during online information dissemination by fixing the transmission rate from the S (Susceptible) state upon contact with the I-state, thereby exploring the superiority of the SSEIR model.

**Figure 10:**
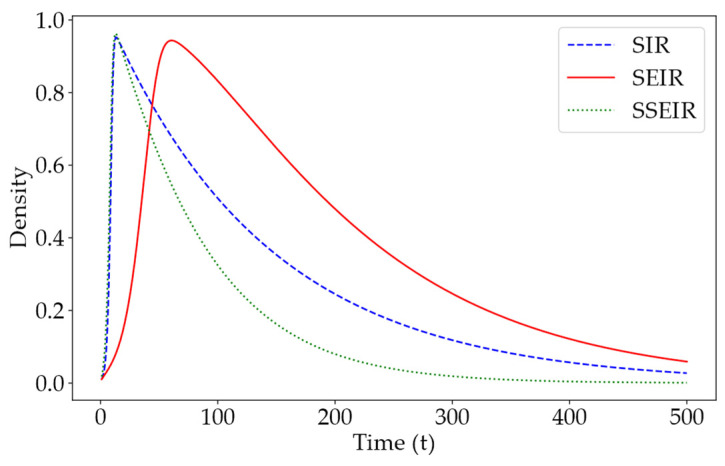
Time-dependent curves of active users in different information dissemination models at a fixed transmission rate. The blue curve in the figure represents the trend in the number of users in the I-state in the SIR model, the red curve represents the trends in the number of users in both the E-state and I-state in the SEIR model, and the green curve represents the trends in the number of users in the E-state and I-state in the SSEIR model.

From Figure 10, it can be observed that at a fixed information transmission rate, the SIR model and the SSEIR model are highly consistent in terms of the peak and timing of active users, reflecting the rapid burst-like spread characteristic of online information networks. However, in the decline phase of information, compared to the slow decay in the SIR model on social networks, the SSEIR model exhibits a faster decay rate. Moreover, the SEIR model significantly lags behind the other two models in both the outbreak and decay phases, indicating that it does not effectively reflect the characteristics of information dissemination in online social networks.

From a structural perspective, compared to the SIR model, the SSEIR model introduces an intermediate state (E-state) for users who have received but not yet disseminated information, allowing for a more precise categorization of users in the information dissemination process. Although the SEIR model recognizes the importance of the intermediate state, it fails to consider that not all users need to pass through the E-state to transition to the dissemination state (I-state). Additionally, the SEIR model is somewhat limited in its design of state transitions, typically allowing only fixed sequences between states.

In real social networks, the transitions between user states are often random and complex. Consequently, in the SSEIR model, we introduced pathways for direct transitions from the E-state to the R-state, as well as from the S-state to the I-state. Overall, based on dual analyses of experimental data and model construction, the SSEIR model demonstrates greater advantages in describing the process of information dissemination in online social networks compared to traditional models.

### 4.8. Validation of the SSEIR Model on Real Datasets

To validate the accuracy of the model on real datasets, we conducted simulation experiments on scientific collaboration networks and real social network datasets. Specifically, the analysis of the scientific collaboration network is based on the High-Energy Physics Phenomenology collaboration network from the large network dataset provided by Stanford University. We posit that the dissemination of academic information among authors shares similarities with information propagation in social networks. By processing the raw data, we associated all authors of the same paper with a hyperedge, where nodes represent the authors. This construction of a hypergraph-based scientific collaboration network more accurately reflects the actual research collaboration relationships. For the social network data, we analyzed a Twitter social network dataset collected by Benedek et al. [40] and others in 2021. Similarly, we processed the social relationship dataset, constructing hyperedges for pairs of connected users, thereby forming a hypergraph-based Twitter social hypernetwork.

**Figure 11:**
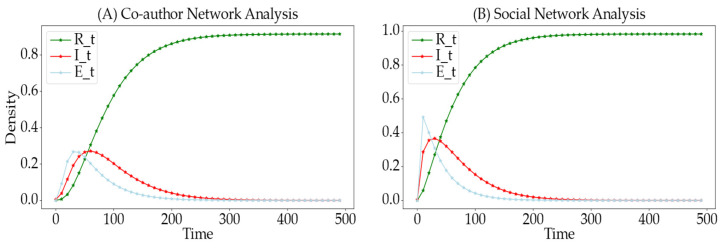
Change curves of different states of the SSEIR model under various real networks. (**A**) shows the validation of the SSEIR model in a scientific collaboration network, while (**B**) depicts the validation in a Twitter social network. The figures use green, red, and blue curves to represent the change curves of the R-state, I-state, and E-state, respectively.

In Figure 11, we use scientific collaboration networks and Twitter social networks to validate the performance of the SSEIR model in real network environments. Through analysis, we find that the trends in the state curves shown in the figure highly align with theoretical predictions and simulation results of generated networks, thereby confirming the effectiveness of the SSEIR model. Comparing Figure 11A and Figure 11B, it is evident that the rates of change in the infected I-state and exposed E-state are significantly faster in the Twitter social network than in the scientific collaboration network. This phenomenon is related to the structural density of the two networks. As concluded in the analysis in Section 4.5, the Twitter social network is more densely structured, resulting in a faster spread of information and quicker immunity acquisition by users. This further validates the effectiveness of our model and the scientific rigor of our experimental methods.

## 5. Conclusion and Discussion

This paper aims to explore the patterns of information dissemination within online social networks and address the deficiencies of traditional models, particularly their inability to classify users and reflect the clustering relationships among them accurately. By utilizing hypernetworks to model the clustering relationships in online social networks and building on the traditional SEIR model of information dissemination, this study introduces the SSEIR model, which incorporates the behavioral characteristics of user nodes within online social networks. Compared to traditional models, the SSEIR model more accurately simulates the real process of information dissemination within the domain of online social networks. The model defines five node states: inactive ($S_{i}$), active ($S_{a}$), exposed (E), informed (I), and recovered (R). Furthermore, we define the rules for state transitions between nodes within the SSEIR model and describe the evolution of these five types of state nodes over time using differential equations. This approach reflects the impact of user behavior characteristics and network parameters on the information dissemination process. Alongside the reaction process strategy, a series of simulation experiments were conducted to validate the accuracy of the theoretical formulas derived in this paper. Furthermore, comparative experiments were conducted to demonstrate the superior capabilities of the hypergraph model over traditional complex network models in characterizing relationships among users of online social networks. This research extensively examined the influence of transmission rates, recovery rates, and network structures on the process of information dissemination. By comparing the SSEIR model to traditional models, the study validated the new model’s enhanced effectiveness in analyzing the dynamics of information dissemination within online social networks. The model’s applicability and scientific precision were further corroborated by testing it in real-world social network environments using publicly available datasets.

This paper introduces a new hypergraph-based model for studying the intricate laws of information dissemination in online social networks, significantly expanding the practical applications and theoretical understanding of hypernetworks in this field. While the current model employs a k-uniform hypergraph, the variability in user clusters in real-world social networks suggests a need for exploring information dissemination on non-uniform hypergraphs, which may offer new insights into user interactions and group dynamics. Considering the dynamic nature of user relationships in online social networks, future research should focus on adapting this model to dynamic hypernetworks to better capture the evolving patterns of user interactions and information flow. Additionally, the considerable variation in how users perceive and react to information calls for more detailed studies into personalized dissemination mechanisms that consider user behaviors and information characteristics. Exploring these research directions will not only deepen our understanding of the complex processes of information dissemination but also provide a robust theoretical foundation for the refinement and enhancement of hypergraph-based models, particularly in predicting and managing online behaviors. Future studies could also extend to applications like social media sentiment monitoring and misinformation control, leveraging the unique strengths of hypergraphs.

## Figures and Tables

**Table 1:** Definition of Node State Transition Probabilities.

Transition	Description
$P_{S_{i}S_{i}}^{i}$	Probability that node i$\;\mathrm{remains}\;\mathrm{in}\;\mathrm{the}\;S_{i}$-state
$P_{S_{i}E}^{i}$	Probability that node i$\;\mathrm{transitions}\;\mathrm{from}\;S_{i}$-state to E-state
$P_{S_{a}S_{a}}^{i}$	Probability that node i$\;\mathrm{remains}\;\mathrm{in}\;\mathrm{the}\;S_{a}$-state
$P_{S_{a}I}^{i}$	Probability that node i$\;\mathrm{transitions}\;\mathrm{from}\;S_{a}$-state to I-state
$P_{EE}^{i}$	Probability that node i remains in the E-state
$P_{EI}^{i}$	Probability that node i transitions from E-state to I-state
$P_{ER}^{i}$	Probability that node i transitions from E-state to R-state
$P_{II}^{i}$	Probability that node i remains in the I-state
$P_{IR}^{i}$	Probability that node i transitions from I-state to R-state

## Data Availability

The data and code used in this work can be accessed via: https://github.com/HaiBingXiao/Dynamical-Analysis-of-an-SSEIR-Information-Propagation-Model-based-on-Hypergraph-in-Social-Networks.git, accessed on 27 September 2024.
